# Glucose-regulated protein 94 deficiency induces squamous cell metaplasia and suppresses PTEN-null driven endometrial epithelial tumor development

**DOI:** 10.18632/oncotarget.7450

**Published:** 2016-02-17

**Authors:** Jieli Shen, Lijing Yao, Yvonne G. Lin, Francesco J. DeMayo, John P. Lydon, Louis Dubeau, Amy S. Lee

**Affiliations:** ^1^ Department of Biochemistry and Molecular Biology, USC Norris Comprehensive Cancer Center, Keck School of Medicine, University of Southern California, Los Angeles, CA 90089, USA; ^2^ Division of Gynecologic Oncology, Department of Obstetrics-Gynecology, USC Norris Comprehensive Cancer Center, Keck School of Medicine, University of Southern California, Los Angeles, CA 90089, USA; ^3^ Department of Molecular and Cellular Biology, Baylor College of Medicine, Houston, TX 77030, USA; ^4^ Department of Pathology, USC Norris Comprehensive Cancer Center, Keck School of Medicine, University of Southern California, Los Angeles, CA 90089, USA

**Keywords:** endometrial cancer, PTEN, glucose-regulated protein 94 (GRP94), squamous cell metaplasia, β-catenin

## Abstract

Endometrial carcinoma is the most prevalent gynecologic cancer in the United States. The tumor suppressor gene *Pten* (phosphatase and tensin homolog) is commonly mutated in the more common type 1 (endometrioid) subtype. The glucose-regulated protein 94 (GRP94) is emerging as a novel regulator for cancer development. Here we report that expression profiles from the Cancer Genome Atlas (TCGA) showed significantly increased *Grp94* mRNA levels in endometrial tumor *versus* normal tissues, correlating with highly elevated GRP94 protein expression in patient samples and the requirement of GRP94 for maintaining viability of human endometrioid adenocarcinoma (EAC) cell lines. Through generation of uterus-specific knockout mouse models with deletion of *Grp94* alone (*c94^f/f^*) or in combination with *Pten* (*cP^f/f^*94*^f/f^*), we discovered that *c94^f/f^* uteri induced squamous cell metaplasia (SCM) and reduced active nuclear β-catenin. The *cP^f/f^*94*^f/f^* uteri showed accelerated SCM and suppression of PTEN-null driven EAC, with reduced cellular proliferation, attenuated β-catenin signaling and decreased AKT/S6 activation in the SCM. In contrast to single PTEN knockout uteri (*cP^f/f^*), *cP^f/f^*94*^f/f^* uteri showed no decrease in E-cadherin level and no invasive lesion. Collectively, our study implies that GRP94 downregulation induces SCM in EAC and suppresses AKT/S6 signaling, providing a novel mechanism for suppressing EAC progression.

## INTRODUCTION

Endometrioid adenocarcinoma (EAC), the most common gynecological malignancy in the United States, arises from the inner lining of the uterus [1]. EAC has been categorized into two distinct types. Type 1 tumors exhibit endometrioid differentiation and account for approximately 85% of the cases, whereas type 2 tumors, non-endometrioid, often display serous or clear cell histology [2]. The most common mutation in type 1 tumors is in the *Pten* tumor suppressor gene which is altered in 40 to 80% of cases [2]. An endometrial cancer mouse model mimicking human EAC has been established using the progesterone receptor promoter driven Cre-recombinase (*PR-Cre*) to conditionally knockout *Pten* in the endometrium, resulting in rapid onset of EAC at 4 weeks of age, with infiltration into the myometrium by 8 weeks [3]. Thus, this model is useful for studying the key determinants of EAC development and invasion.

Glucose-regulated protein 94 (GRP94) encoded in humans by *HSP90B1,* is a member of the HSP90 family sharing 50% homology to cytosolic paralog HSP90 [4, 5]. It is a multifunctional protein playing important roles as a molecular chaperone and a Ca^2+^-binding protein in the endoplasmic reticulum (ER) [6, 7]. Homozygous knockout of GRP94 results in embryonic lethality [8, 9]. As an ER chaperone, GRP94 controls the maturation and secretion of insulin growth factors (IGFs) and the processing of Toll-like receptors [7, 10]. Recently, the Wnt co-receptor, LRP6, has been identified as a new client of GRP94 [11]. When GRP94 was conditionally knocked out in the gut, LRP6 failed to export to the cell surface, and nuclear translocation of β-catenin was compromised [11]. As the β-catenin pathway is a key regulator of development as well as tumorigenesis, this suggests that loss of GRP94 could impact cellular differentiation and cancer progression. Invasive cancer requires remodeling of the cell adhesion molecules that maintain cell-cell contacts and intercellular junctions [12]. Another major function attributed to GRP94 is in maintaining cell matrix integrity due to its chaperoning activities for key cell surface adhesion proteins such as E-cadherin and integrins [13–15].

GRP94 is commonly elevated in human cancers [5]. The *Grp94* promoter was strongly activated in spontaneous and chemically-induced tumors in mice [16]. GRP94 loss in B cells attenuated multiple myeloma, and GRP94 deficiency in macrophages reduced colitis and inflammation-associated colon tumorigenesis [17, 18]. However, in the bone marrow and hepatocytes, GRP94 loss perturbed extracellular matrix proteins, oncogenic signaling pathways and led to hyper-proliferation of progenitor/stem cells [13, 14]. Interestingly, repopulation of GRP94-positive hepatocytes was detected in GRP94-deficient livers, correlating with spontaneous hepatocellular carcinoma development in aged mice and promotion of chemically-induced hepatocellular carcinogenesis [19, 20]. Therefore, GRP94 could have a multifaceted effect on tumorigenesis that is context- and age-dependent. In this study, we report that GRP94 expression is significantly elevated in human endometrial cancer and required for human endometrial cancer cell viability. We further report the creation of two endometrial-specific knockout mouse models with deletion of *Grp94* alone or in combination with *Pten* deletion. Our study reveals that loss of GRP94 in mouse endometrium induces squamous cell metaplasia (SCM), attenuated PTEN-deficiency mediated β-catenin and AKT/S6 activation in the SCM and suppressed EAC.

## RESULTS

### GRP94 mRNA and protein levels are elevated in human EAC and contribute to cell viability

**Figure 1:**
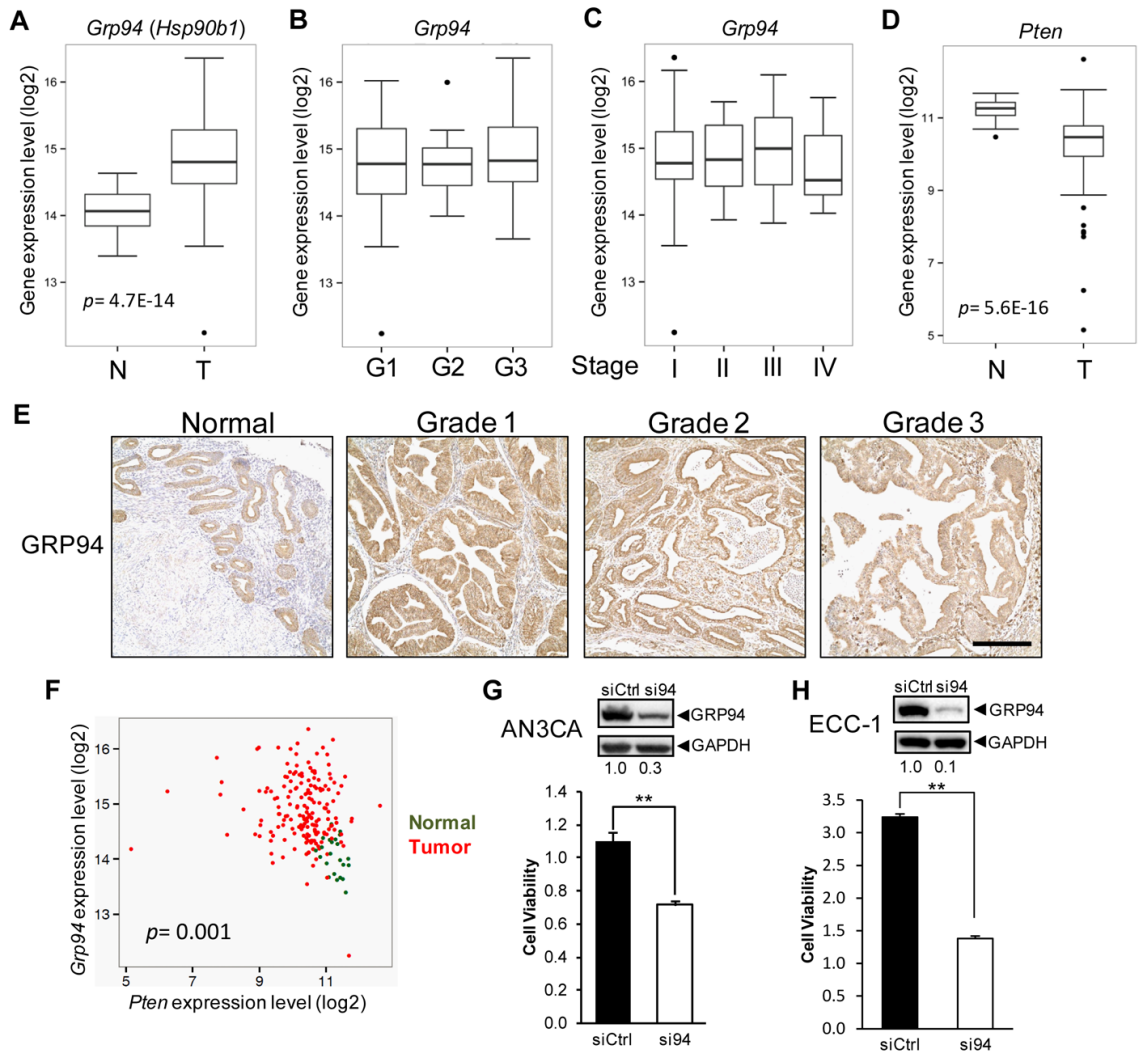
Increased GRP94 expression in human EAC and its effect on EAC cell viability *Grp94* mRNA expression in **A.** normal uterine (n=24) and EAC tissues (n=177), **B.** different grade EAC, and **C.** different stage EAC. **D.** *Pten* mRNA expression in normal uterine and EAC tissues. Student's *t*-test *p* values are indicated. **E.** Immunohistochemistry (IHC) analysis of GRP94 in human normal uterine and EAC tissues. Scale bar, 50 μm. **F.** Scatterplot of *Grp94* and *Pten* mRNA expression of human normal uterine (green) and tumor samples (red). Pearson correlation *p* value is indicated. Western blot analysis of GRP94 knockdown efficiency in **G.** AN3CA cells and **H.** ECC-1 cells. The level of GRP94 reduction after normalization to GAPDH which served as loading control is shown below. WST-1 assay measuring cell viability in si-control (siCtrl) and si-Grp94 (si94) treated **G.** AN3CA cells at day 4 and **H.** ECC-1 cells at day 5. Data are presented as mean ± s.e., **p<0.01 (Student's *t*-test).

First, we performed *Grp94* mRNA expression analyses using 24 normal and 177 endometrial cancer expression profiles from TCGA [21]. We observed significantly increased *Grp94* mRNA expression in tumor *versus* normal samples (*p* value = 4.7E-14), and comparable *Grp94* mRNA levels in different grade and stage of endometrial cancer (Figures 1A-1C). As many *Pten* mutations result in decreased expression [2, 22], *Pten* mRNA is downregulated in tumor samples compared to normal samples (*p* value = 5.6E-16) (Figure 1D). Furthermore, examination of GRP94 protein expression in human uterine tissue samples by immunohistochemistry (IHC) showed basal GRP94 expression in epithelial cells in the normal endometrium, and upon development of EAC which is populated by epithelial cells, strong GRP94 expression was detected uniformly in Grade 1, 2 and 3 tumors (Figure 1E), in agreement with the RNA-seq dataset analysis. Furthermore, consistent with GRP94 over-expression in endometrial cancer which is predominantly mutated in *Pten*, in RNA-seq dataset analysis, an inverse correlation was detected between *Grp94* and *Pten* mRNA levels using both normal (green) and tumor samples (red) (*p* value = 0.001) due to the opposite expression changes between *Pten* and *Grp94* mRNA levels from normal to tumor (Figure 1F).

To test the effect of GRP94 deficiency on the viability of human endometrial cancer cells, two cell lines (AN3CA and ECC-1), both reported to have loss of PTEN [23], were examined. Treatment with siRNA targeting *Grp94* reduced its protein level in AN3CA cells by 70% and in ECC-1 cells by 90% compared to control siRNA (siCtrl), corresponding with a 35% and 60% decrease in viability of AN3CA and ECC-1 cells, respectively, compared to siCtrl, as measured by the WST-1 assay (Figures 1G and 1H). Thus, GRP94 contributes to human endometrial cancer cell viability.

### Endometrial GRP94 ablation leads to spontaneous squamous cell metaplasia and loss of active nuclear β-catenin expression

**Figure 2:**
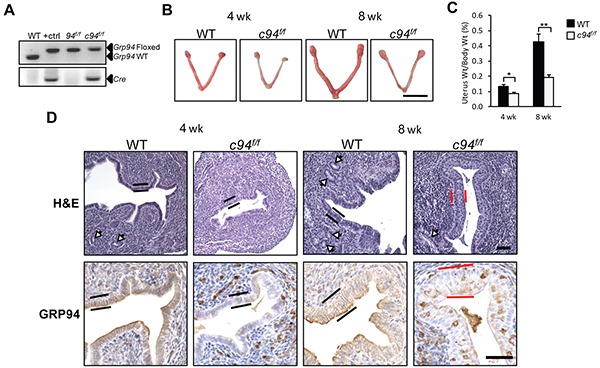
Generation of *PR-Cre*-mediated GRP94 knockout mouse models **A.** Representative mouse tail PCR genotyping of the indicated alleles. **B.** Representative gross anatomy of uteri of the indicated genotypes at 4 and 8 weeks. Scale bar, 1 cm. **C.** The ratio of uterine weight to body weight in WT and *c94^f/f^* mice at 4 weeks (n=16 and 5, respectively) and 8 weeks (n=4 and 11, respectively). The data are presented as mean ± standard error (s.e.), *p<0.05, **p<0.01 (LSD Method, and data was log transformed prior to analysis). **D.** H&E staining (upper panel) and IHC analysis of GRP94 (lower panel) of WT and *c94^f/f^* uteri from 4 and 8 weeks. Scale bar, 50 μm. White arrows indicate glands and black and red bars denote columnar luminal epithelial cells and SCM, respectively.

To study the role of GRP94 in endometrial cancer *in vivo*, we utilized genetically engineered mouse models. First, we determined the effect of GRP94 ablation in the normal mouse endometrium. Deletion of GRP94 in the post-natal mouse endometrium was achieved through creation of mice bearing a *PR-Cre* transgene and *Grp94* floxed alleles. The PR promoter is activated in endometrial tissues around post-natal day 3 leading to gene ablation in luminal and glandular epithelial cells at first and subsequently in endometrial stroma and myometrium [24]. The *PR-Cre;Grp94^f/f^* mice, referred to below as *c94^f/f^*, were analyzed in parallel with littermates lacking *PR-Cre* serving as WT controls. The genotypes of the mice were determined by PCR of tail genomic DNA (Figure 2A). At both 4 and 8 weeks, the *c94^f/f^* uteri were smaller (Figure 2B) and lighter (Figure 2C). Histological examination of uterine sections stained with H&E revealed two major differences. First, the *c94^f/f^* uteri exhibited reduced number of endometrial glands compared to WT (Figure 2D, white arrows), resulting in a flat luminal surface. Second, while the WT uteri were lined with a single layer of columnar cells (black bars), at 8 weeks the *c94^f/f^* uteri were lined by multi-layered squamous epithelium (Figure 2D, red bars). In contrast, uteri deficient of another ER chaperone GRP78 did not exhibit SCM and were lined with a single layer of columnar cells (Supplementary Figure S1). As demonstrated by IHC, GRP94 expression was largely depleted in the *c94^f/f^* uteri except in occasional stromal cells (Figure 2D).

**Figure 3:**
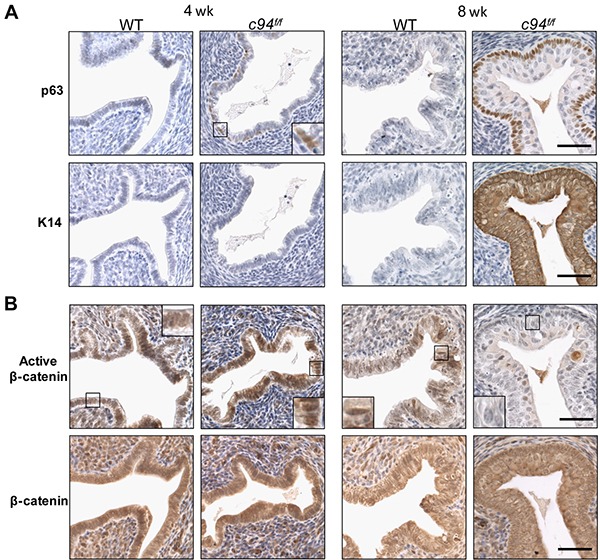
Induction of squamous metaplasia in developing *c94^f/f^* uteri **A.** IHC of p63 and cytokeratin 14 (K14). **B.** IHC of active β-catenin and β-catenin in WT and *c94^f/f^* uteri at 4 and 8 weeks. Enlarged views of the boxed regions are shown. Scale bar, 50 μm.

To confirm that the multi-layered epithelium in *c94^f/f^* uteri represented squamous metaplasia, the expression of p63 and cytokeratin 14 (K14), representing early and late markers of squamous differentiation, respectively, was examined by IHC [25, 26]. At 4 weeks, p63 expression was detected in some *c94^f/f^* endometrial epithelial cells located within the luminal lining, indicating initiation of SCM; by 8 weeks *c94^f/f^* endometrial epithelial cells showed robust p63 and K14 expression, confirming their squamous nature (Figure 3A). As expected, both markers were absent from WT endometrium.

We next tested whether SCM observed in the *c94^f/f^* endometrium could be due to β-catenin dysfunction since ablation of endometrial β-catenin has been reported to induce SCM [27, 28]. IHC analysis revealed that at 4 weeks both *c94^f/f^* and WT endometria showed strong active nuclear β-catenin expression in the columnar epithelium (Figure 3B, box), indicating that GRP94 is not required for β-catenin activation and nuclear translocation in columnar cells. However, 8 week old *c94^f/f^* endometrium, which had undergone SCM, showed minimal active nuclear β-catenin expression compared to WT (Figure 3B, box). Total β-catenin levels were similar in *c94^f/f^* and WT endometria at 4 and 8 weeks (Figure 2B). No spontaneous endometrial cancer was observed in the *c94^f/f^* uteri for at least 9 months (Supplementary Figure S2).

### Endometrial GRP94 deficiency suppresses PTEN-null driven adenocarcinoma

**Figure 4:**
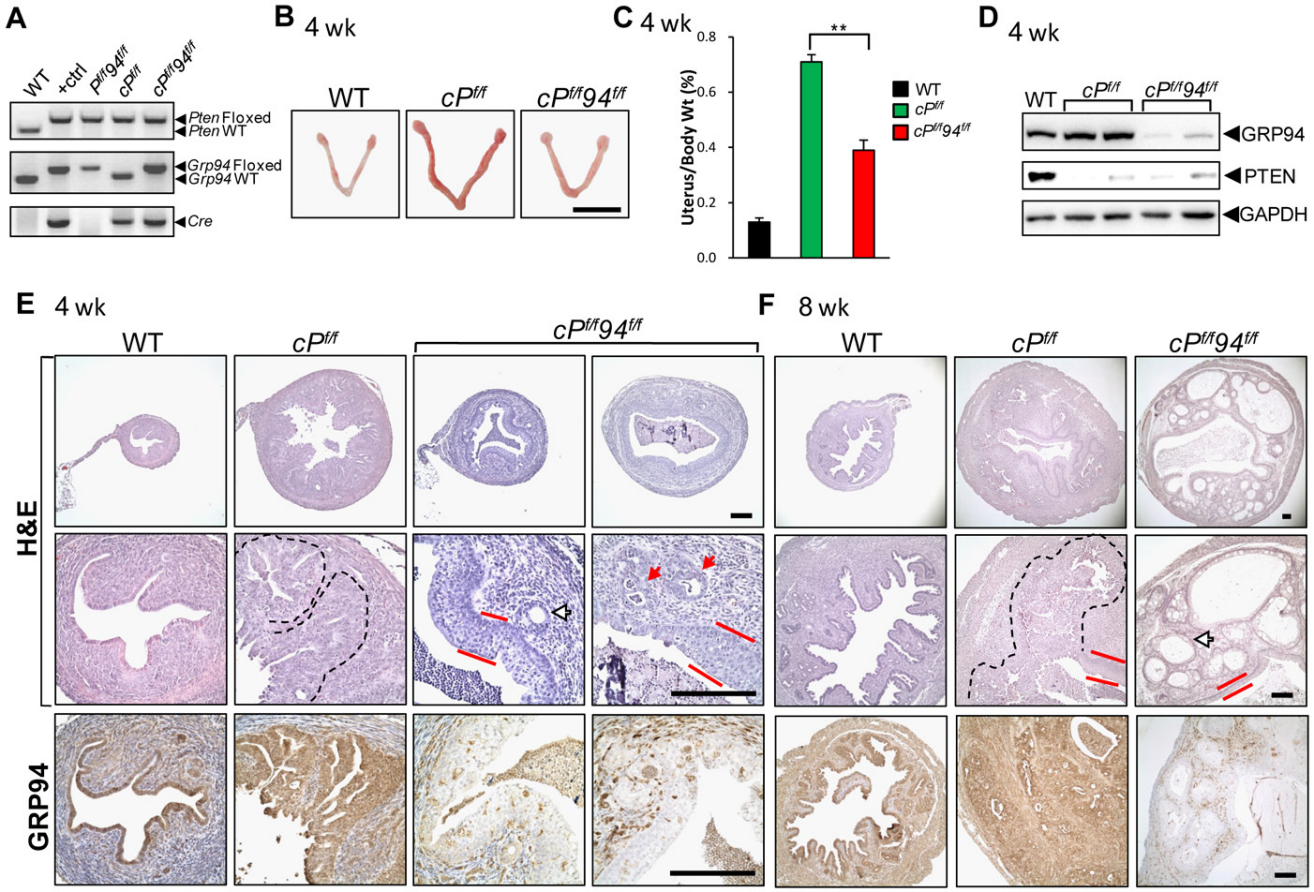
Reduced endometrial cancer in *cP^f/f^*94*^f/f^* uteri **A.** Representative mouse tail PCR genotyping of the indicated alleles. **B.** Representative gross anatomy of uteri of the indicated genotype at 4 weeks. Scale bar, 1 cm. **C.** The ratio of uterine weight to body weight in WT (n=16), *cP^f/f^* (n=10) and *cP^f/f^*94*^f/f^* (n=9) mice at 4 weeks. The data are presented as mean ± s.e., **p<0.01 (LSD Method). **D.** Western blot analysis of GRP94 and PTEN levels in uteri of indicated genotypes with GAPDH serving as the loading control. **E.** H&E staining and IHC of GRP94 in uteri of the indicated genotype at 4 weeks and **F.** at 8 weeks. White and red arrows indicate normal and transformed glands, respectively, the broken lines denote EAC and red bars denote SCM. Scale bar, 200 μm.

To test the role of GRP94 in type 1 endometrial cancer, which can be induced by PTEN depletion, *PR-Cre;Pten^flox/flox^* (*cP^f/f^*) mice were crossed with *Pten^f/f^*94*^f/f^* mice to generate a biallelic deletion strain lacking both *Pten* and *Grp94* (*cP^f/f^*94*^f/f^*). Littermates lacking the *PR-Cre* transgene were used as WT controls. The mouse genotypes were determined by PCR of tail genomic DNA (Figure 4A). At 4 weeks, the *cP^f/f^* uteri were considerably larger than the WT and *cP^f/f^*94*^f/f^* uteri (Figure 4B), and the average weight of *cP^f/f^* uteri normalized against body weight was 5.5 and 2.0 times that of the WT and *cP^f/f^*94*^f/f^* uteri, respectively (Figure 4C). Western blot analysis of uterine tissue lysates confirmed minimal expression of PTEN and GRP94 in the respective knockout mice, with GAPDH serving as loading control (Figure 4D).

Histological examination showed that at 4 weeks all 9 *cP^f/f^* uteri examined contained extensive and aggressive EAC which, in 8 of the 9 cases, replaced more than 70% of the endometrium (Figure 4E). One out of 7 *cP^f/f^*94*^f/f^* uteri showed no evidence of malignancy. The remaining 6 *cP^f/f^*94*^f/f^* uteri showed a mixture of normal and transformed glands (Figure 4E, white and red arrows, respectively). Five out of 6 showed 30% or less of the endometrium replaced by tumor, while only one uterus showed more extensive tumor involvement. In addition, all tumors seen in *cP^f/f^*94*^f/f^* uteri were separated from the myometrium by a layer of normal endometrium, as opposed to the tumors from *cP^f/f^* uteri that typically involved the full thickness of the endometrium and abutted the myometrium. All *cP^f/f^*94*^f/f^* uteri showed extensive squamous metaplasia of the endometrial luminal lining (Figure 4E, red bars). At 8 weeks, *cP^f/f^* uteri continued to show aggressive EAC, and SCM of the luminal epithelium was detected (Figure 4F). Strikingly, 8 week old *cP^f/f^*94*^f/f^* uteri only showed low grade lesions characterized by large well-differentiated glandular structures with no morphological features of invasiveness such as ragged edges or stromal reactions (Figure 4F). GRP94 depletion in both 4 and 8 week *cP^f/f^*94*^f/f^* uteri, including in the well-differentiated glandular structures, was confirmed by IHC (Figures 4E and 4F).

### *cP^f/f^*94*^f/f^* uteri display early squamous metaplasia and reduced β-catenin signaling

**Figure 5:**
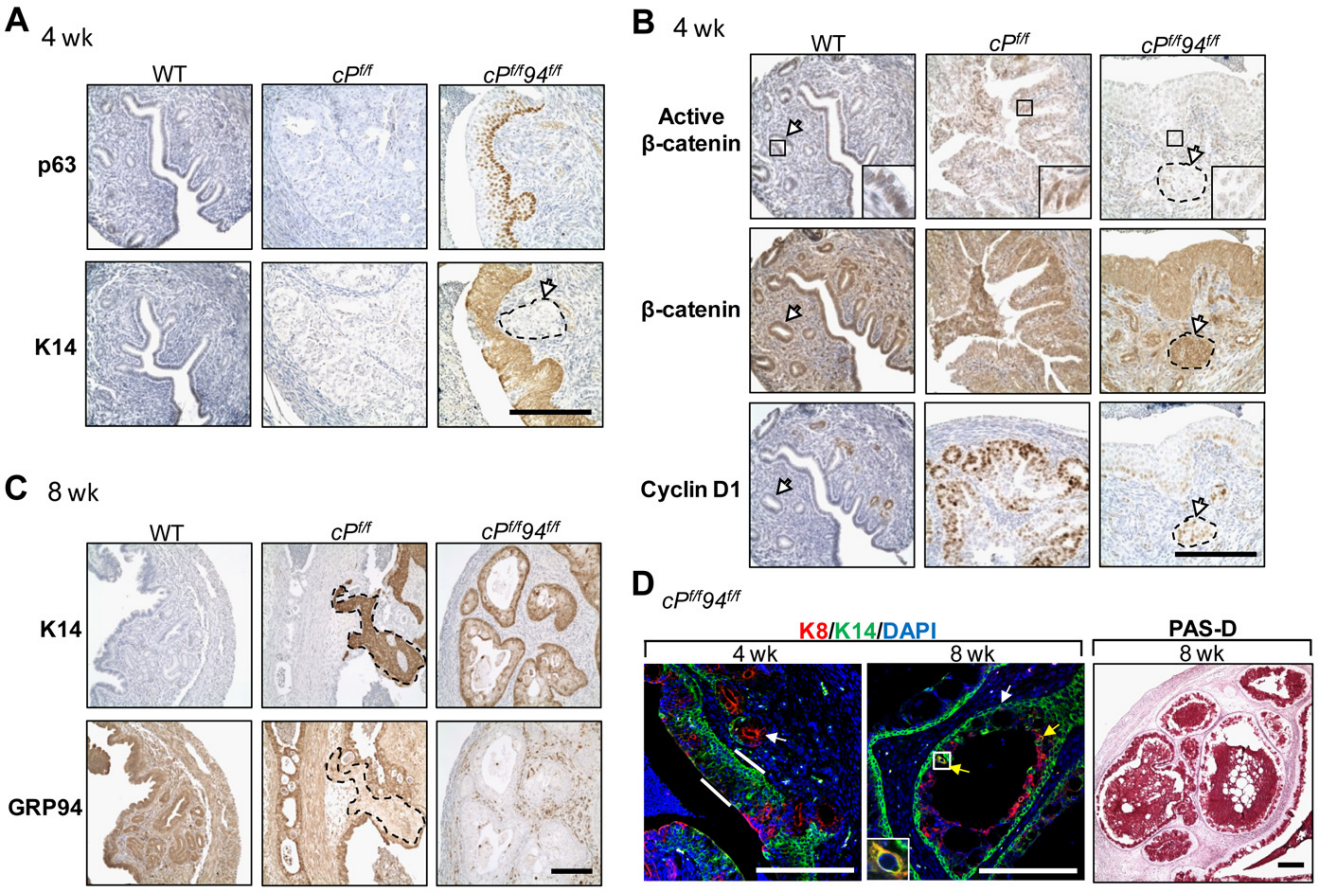
Accelerated squamous metaplasia and reduced β-catenin signaling in *cP^f/f^*94*^f/f^* uteri **A.** IHC of p63, K14 and **B.** IHC of active β-catenin, β-catenin, cyclin D1 in uteri of indicated genotypes at 4 weeks. The white arrows denote glands. Enlarged view of the boxed region is shown. **C.** IHC of K14, GRP94 in uteri of indicated genotypes at 8 weeks. Scale bar, 200 μm. **D.** Merged images of immunofluorescence (IF) of K8 (red) and K14 (green) with nuclei stained with DAPI (blue) in *cP^f/f^*94*^f/f^* uteri and PAS-diastase staining of *cP^f/f^*94*^f/f^* uteri at the indicated age. Scale bar, 200 μm. White arrows indicate glands, white bars denote SCM and yellow arrows indicate cells positive for both K8 and K14. Enlarged view of the boxed region is shown.

Through IHC, we observed luminal epithelial cells were positive for both p63 and K14 at 4 week old *cP^f/f^*94*^f/f^* uteri (Figure 5A), indicating accelerated onset of SCM compared to age-matched *c94^f/f^* and *cP^f/f^* uteri. Active nuclear β-catenin expression was readily detected in the WT and *cP^f/f^* but not the *cP^f/f^*94*^f/f^* uteri while total β-catenin was robustly expressed in all three genotypes (Figure 5B). As a critical transcription factor, nuclear β-catenin activates downstream targets such as cyclin D1 which induces progression through the G1 phase of the cell cycle [29]. Expression of cyclin D1 was more prominent in the transformed glandular structures in *cP^f/f^* uteri compared to WT epithelium attesting to increased cell cycle activity (Figure 5B). Expression of cyclin D1 was low and confined to the basal layers in the metaplastic epithelium of *cP^f/f^*94*^f/f^* uteri, indicating an ordered proliferation and a level of organization characteristic of untransformed squamous epithelium (Figure 5B). At 8 weeks, IHC for K14 confirmed that PTEN-null uteri showed SCM in some areas and, interestingly, those same regions showed low GRP94 expression (Figure 5C, broken lines). Thus, both *c94^f/f^* and *cP^f/f^* uteri showed squamous differentiation, correlating with GRP94 deficiency. We compared the distribution of metaplastic squamous cells (K14 positive) *versus* columnar cells (K8 positive) in *cP^f/f^*94*^f/f^* uteri. Co-staining for K14 (green) and K8 (red) showed that at 4 weeks, whereas endometrial glands were K8 positive (white arrow), most of the endometrial luminal cells expressed the marker of squamous differentiation (K14) with some residual cells located in the top layer (luminal surface) expressing K8 (Figure 5D). By 8 weeks, both endometrial luminal and glandular cells expressed the squamous marker (K14), with some cells expressing glandular marker (K8) located near the glandular lumen. Occasional cells expressed both K14 and K8 markers (Figure 5D, yellow arrows and box). Intra- and extra-cellular mucin, indicative of secretory activity, was present in 8 week old *cP^f/f^*94*^f/f^* uteri, as confirmed by PAS-diastase (Figure 5D) and mucicarmine (not shown) positivity.

### Attenuation of AKT/S6 activation and proliferation in *cP^f/f^*94*^f/f^* SCM

**Figure 6:**
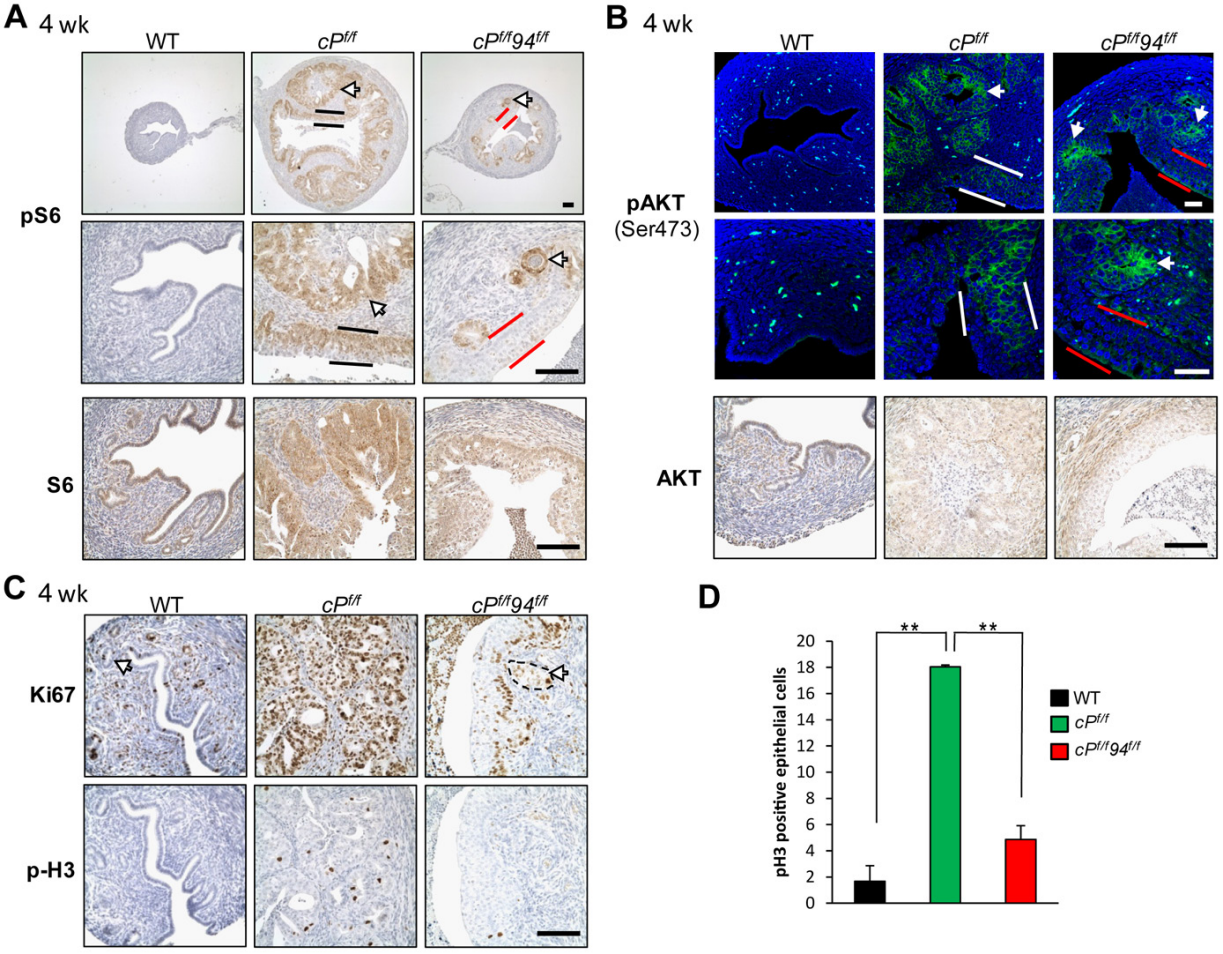
Attenuated AKT activation and decreased proliferation in *cP^f/f^*94*^f/f^* SCM **A.** IHC of phospho-S6 (pS6) and S6 in uteri of the indicated genotypes at 4 weeks. White arrows indicate glands, black and red bars denote columnar luminal epithelial cells and SCM, respectively. **B.** IF of pAKT (Ser 473) and IHC of AKT. White arrows indicate glands, white and red bars denote columnar luminal epithelial cells and SCM, respectively. **C.** IHC of Ki67 and phospho-Histone H3 (p-H3) in mouse uteri. Scale bar, 100 μm. **D.** Quantification of the p-H3 positive epithelial cells in the uteri sections of WT (n=6), *cP^f/f^* (n=3) and *cP^f/f^*94*^f/f^* (n=4) uteri at 4 weeks. The data are presented as mean ± s.e., **p<0.01 (LSD Method).

*Pten* deletion leads to PI3K signaling with activation of downstream targets including AKT and S6 via phosphorylation [30]. As expected, *cP^f/f^* uteri showed robust activation of S6 in the EAC (Figure 6A, white arrows) and luminal epithelial cells (black bars), whereas in *cP^f/f^*94*^f/f^* uteri S6 activation was confined to the glands but absent in the SCM (Figure 6A, red bars). Immunofluorescence (IF) analysis for activated AKT showed a pattern similar to that of pS6 in the three genotypes such that AKT phosphorylation was evident in the glands of *cP^f/f^* and *cP^f/f^*94*^f/f^* uteri (white arrows), but substantially reduced in the SCM of the *cP^f/f^*94*^f/f^* uteri (Figure 6B, red bars) compared to the columnar luminal epithelial cells of *cP^f/f^* uteri (white bars). IHC analysis showed no ERK activation in uteri of all three genotypes (Supplementary Figure S3). Proliferation as measured by IHC markers expressed either throughout the cell cycle (Ki67) or during the mitotic phase (phospho-histone H3), was substantially reduced in the *cP^f/f^*94*^f/f^* compared to *cP^f/f^* uteri and was confined to glandular cells and basal layer of SCM (Figures 6C and 6D). We investigated the potential contribution of apoptosis to the reduced size of *cP^f/f^*94*^f/f^* uteri. IHC showed that whereas *cP^f/f^* uteri exhibited a few cells positive for cleaved caspase-3 located towards the lumen, *cP^f/f^*94*^f/f^* uteri showed little if any cells positive for cleaved caspase-3 (Supplementary Figure S4). Collectively, these results indicate that suppression of AKT/S6 activation and reduced proliferation, rather than leading to increased apoptosis, likely contribute to the suppression of PTEN-null driven EAC in *cP^f/f^*94*^f/f^* uteri.

### *cP^f/f^*94*^f/f^* uteri show no EAC myometrial invasion and high E-cadherin expression

**Figure 7:**
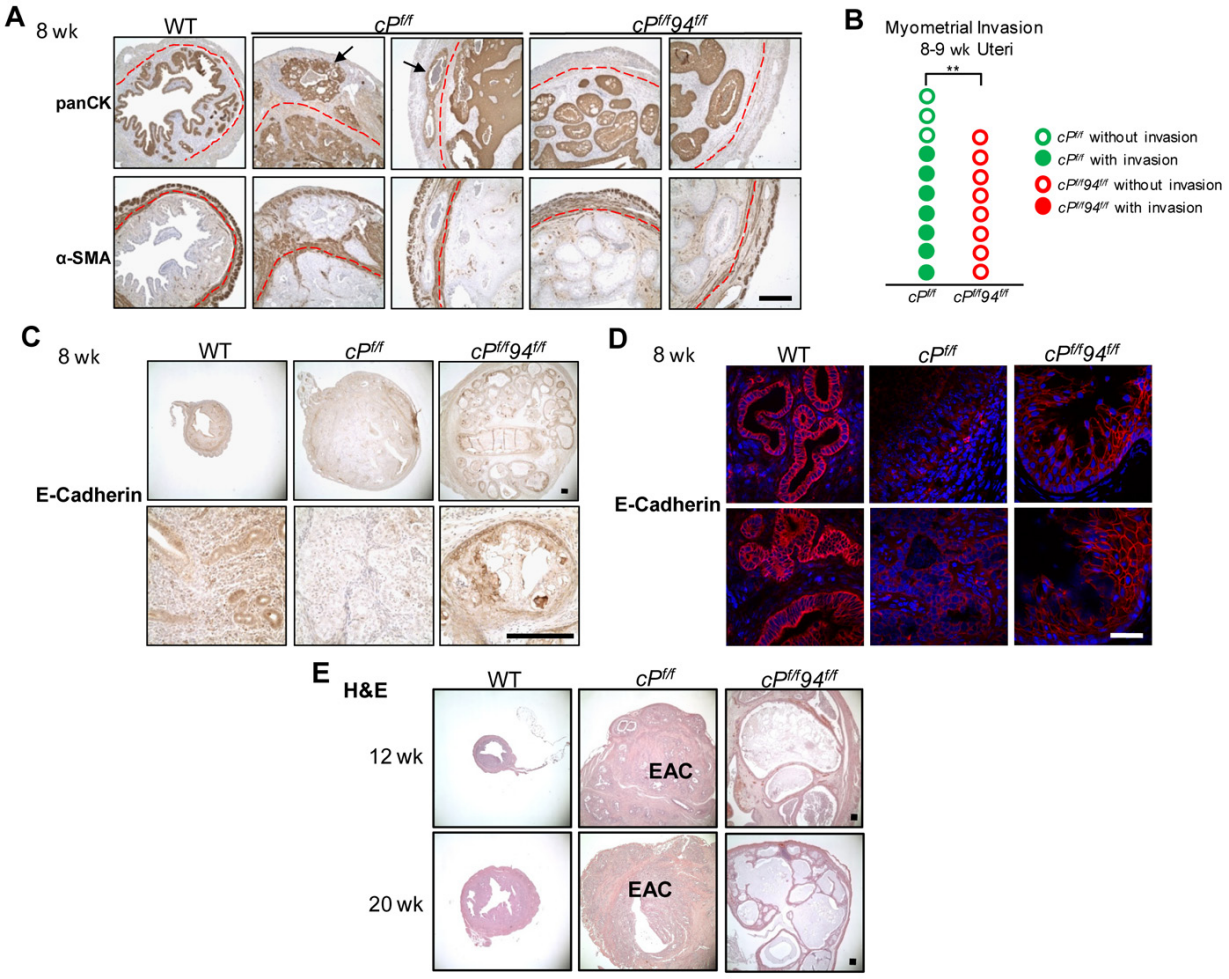
Characterization of *cP^f/f^*94*^f/f^* uteri on myometrial invasion and prolonged stage **A.** IHC of pan-cytokeratin (panCK) and α-smooth muscle actin (α-SMA) on consecutive slides in uteri of the indicated genotypes at 8 weeks. The red broken lines denote the boundaries between the endometrium and the myometrium. Arrows indicate EAC invasion into the myometrium. Scale bar, 400 μm. **B.** Frequency of myometrial invasion in *cP^f/f^* and *cP^f/f^*94*^f/f^* uteri at 8-9 weeks. Each circle represented one mouse. *p*=0.004 (2-sided Fisher's exact test). **C.** IHC of E-cadherin at 8 weeks. Scale bar, 200 μm. **D.** IF of E-cadherin on frozen tissue sections (red) at 8 weeks. Nuclei were stained with DAPI (blue). Scale bar, 50 μm. **E.** H&E of WT, *cP^f/f^* and *cP^f/f^*94*^f/f^* uteri at 12 weeks (upper panel) and 20 weeks (lower panel). Scale bar, 200 μm.

We next compared myometrial invasion of *cP^f/f^* and *cP^f/f^*94*^f/f^* uteri through histological examination of H&E stained tissue sections by a pathologist (L.D.), as well as examination of IHC stains on consecutive microtome sections for a muscle layer marker, α-smooth muscle actin (α-SMA) and an epithelial cell marker, pan-cytokeratin (panCK) [3]. Myometrial invasion was readily observed in *cP^f/f^* uteri at 8 weeks (Figure 7A, arrows). In contrast, no myometrial invasion was detected in age-matched *cP^f/f^*94*^f/f^* uteri (Figure 7A). Seven of 10 *cP^f/f^* uteri exhibited myometrial invasion at that age compared to 0 out of 8 *cP^f/f^*94*^f/f^* uteri (Figure 7B). These differences were statistically significant (*p* = 0.004). Consistent with this notion that invasive cancer cells loosen their connection to neighboring cells and basement membrane via downregulation of intercellular adhesion [12], *cP^f/f^* uteri in which myometrial invasion was present, exhibited very low level of E-cadherin (Figures 7C and 7D). In contrast, both WT and *cP^f/f^*94*^f/f^* uteri showed high E-cadherin expression in epithelial cells.

### Prolonged depletion of GRP94 in PTEN-null uteri leads to massive glandular expansion but no EAC formation

**Figure 8:**
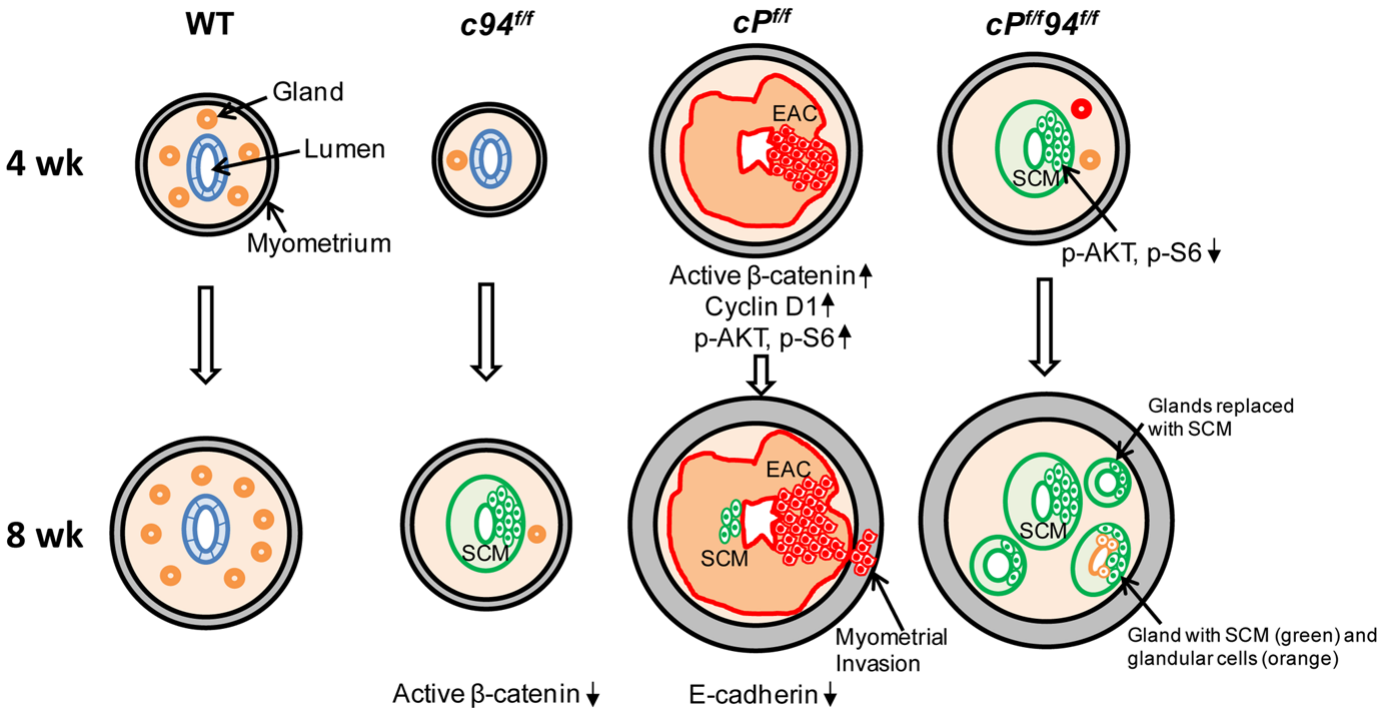
Summary model on the effect of GRP94 deficiency in mouse uteri in the presence or absence of PTEN-deficiency The development of 4 and 8 week uteri of the indicated genotypes is shown. The morphological transitions of the glands, SCM replacement, EAC formation, alteration of the signaling pathways and E-cadherin expression are summarized for 4 and 8 week uteri. EAC: endometrial cancer. SCM: squamous cell metaplasia.

In following WT, *cP^f/f^* and *cP^f/f^*94*^f/f^* mice past 8 weeks, we noticed that the size of the *cP^f/f^*94*^f/f^* uteri was considerably larger than the *cP^f/f^* uteri by 12 and 20 weeks (Supplementary Figure S5A). For both 12 and 20 week uteri, histological examination showed aggressive EAC in the *cP^f/f^* uteri, whereas the *cP^f/f^*94*^f/f^* uteri displayed massively expanded, but mature glands with abundant secretions lacking any of the cytological and architectural features typically associated with malignancy (Figure 7E). IHC further showed that GRP94 depletion had persisted in the *cP^f/f^*94*^f/f^* uteri at least through 20 weeks (Supplementary Figure S5B). The effects of GRP94 deficiency in the WT (*c94^f/f^*) and PTEN-null uteri (*cP^f/f^*94*^f/f^*) are summarized in Figure 8, with the striking observation that while PTEN-null uteri with WT GRP94 level all developed EAC by 4 weeks, no EAC was detected in the uteri of 12 to 20 week *cP^f/f^*94*^f/f^* mice.

## DISCUSSION

The glucose regulated proteins including GRP94 and GRP78 are stress-inducible chaperones that mostly reside in the ER and are well-established as molecular chaperones critical for the ER homeostasis [5]. While GRP94 shares partial amino acid homology with HSP90, its cellular localization and functions are distinct from HSP90 [6]. At the same time while GRP94 and GRP78 are coordinately upregulated by ER stress, knockout of either GRP94 or GRP78 in mouse models resulted in embryonic lethality, implying distinct and non-compensatory functions in early mammalian development [8, 31]. Recently we have examined the consequence of GRP78 knockout alone or in combination of PTEN in the mouse uteri and discovered that while both GRP78 and GRP94 deficient uteri were smaller in size and showed a decrease in the number of glands, unlike GRP94, GRP78 deficient uteri were lined with a single layer of columnar cells. The uteri from the *cP^f/f^*78*^f/f^* uteri showed decreased AKT activation and were devoid of atypical hyperplasia, a well-established EAC precursor, throughout the experimental period of up to 8 months [32].

These interesting observations raise the question whether other ER chaperones such as GRP94 could also impact EAC development, and if so, do they utilize the same or different mechanism? Results from the Cancer Genome Atlas analysis in this study provided the first clue that *Grp94* mRNA expression is significantly increased in human EAC tissues across all grades of EAC, confirmed by IHC staining of human EAC samples. Recent evidence also showed that GRP94 suppresses AKT activation in hepatocellular carcinoma *in vivo* and *in vitro* [13, 33], implying that AKT activation in PTEN-null driven EAC may also be impaired by the loss of GRP94. In this study, we investigated the effect of GRP94 deficiency in the mouse uterus in the absence or presence of the tumor suppressor gene *Pten*, which is commonly altered in type 1 endometrial cancer. This study reveals new GRP94 function on uteri development and oncogenic signaling which could offer novel mechanisms for curbing EAC.

First, compared to WT, GRP94 deficient uteri exhibited a decrease in the number of endometrial glands, depriving adenocarcinoma of a substrate from which to develop. This could explain in part the lower incidence of EAC in the *cP^f/f^*94*^f/f^* uteri, as well as the smaller size of such uteri, since budding of nascent glands from the luminal epithelium is critical for uterine development [34]. Loss of GRP94 might also lead to decreased uterine size through IGF1 deficiency, since GRP94 is an essential chaperone for the production of IGFs [7, 9, 35], and IGF-1 deficient mice possess an infantile uterus with severe hypoplasia especially in the myometrium [36].

Strikingly, uterine GRP94 deficiency resulted in spontaneous replacement of the columnar luminal epithelial cells with squamous cells as early as 8 weeks. Such replacement of one differentiated cell type with another, defined as metaplasia, while a rare occurrence in the healthy endometrium, can be observed occasionally and under both physiological and pathological conditions such as endometrial cancer, chronic irritation or progestin effects [37–39]. The reduced number of glands and the presence of SCM in GRP94-deficient uteri resembled the phenotypes associated with uterine β-catenin ablation [27]. Indeed, depletion of uterine GRP94 resulted in loss of active nuclear β-catenin, suggesting that this could contribute in part to spontaneous SCM in the *c94^f/f^* uteri. SCM was accelerated when GRP94 deficiency was superimposed on a PTEN deficiency, which independently induces endometrial SCM [40]. In support of the notion that GRP94 deficiency promotes SCM induction, we observed that the SCM cells in the PTEN-null uteri also expressed low GRP94. Interestingly, uterine depletion of GRP78, in the presence [32] or absence of PTEN-deficiency did not induce SCM (Supplementary Figure S1), underscoring the unique role of GRP94 in SCM.

Our work provides the first evidence that GRP94 deficiency can delay the development of cancers driven by the absence of a functional PTEN. Interestingly, this study revealed that the majority of *cP^f/f^*94*^f/f^* uteri at 4 weeks did develop endometrial cancer albeit the tumors were less aggressive and much smaller in overall volume compared to the EAC seen in the *cP^f/f^* uteri. However, by 8 weeks, the morphological appearance of the *cP^f/f^*94*^f/f^* tumors had become substantially different than that of the tumors seen in the *cP^f/f^* uteri. The lesions were low-grade and less aggressive as evidenced by smooth acinar contours and abundant secretions indicative of higher degree of differentiation. The *cP^f/f^*94*^f/f^* uteri showed WT level of E-cadherin and no sign of myometrial invasion by tumor. We further noted that in 8 week *cP^f/f^*94*^f/f^* uteri, the majority of the cells expressed the squamous marker (K14). Cells expressing glandular marker (K8) were located in the luminal layer, suggesting that the precursor cells normally giving rise to columnar epithelial cells alternatively initiate a different differentiation pathway leading to squamous cells. Beyond 8 weeks, the *cP^f/f^*94*^f/f^* uteri rapidly increased in size and the endometrium showed expanded glands with abundant secretions. The accumulation of secretion could be due either to an effect of GRP94 deficiency on secretory activity or to entrapment of secretions within glandular lumina because of narrowing of secretory ducts due to squamous metaplasia. In summary, loss of uterine GRP94 leads to several unique phenotypes clearly distinguishable from those associated with suppression of EAC development in conditional GRP78 knockout mice [32]. We show that replacing columnar epithelial cells with squamous epithelial cells induced by GRP94 deficiency may represent a novel mechanism for suppression of EAC.

## MATERIALS AND METHODS

### Mice

*PR-Cre;Pten^f/f^* mice on a C57BL6/129SV background [3] were crossed with *Pten^f/f^*;Grp94*^f/f^* mice [13] to generate the *PR-Cre;Pten^f/f^*;Grp94*^f/f^* (*cP^f/f^*94*^f/f^*) mice. *Grp94^f/f^* mice on a C57BL/6;129/SV background [8] were crossed with *cP^f/f^*94*^f/f^* to generate *PR-Cre;Grp94^f/f^* (*c94^f/f^*). Genotyping was performed by PCR using mouse tail genomic DNA as previously described [13]. All protocols for animal use and euthanasia were reviewed and approved by the University of Southern California Institutional Animal Care and Use Committee.

### Tissue processing and histology

Female mice were euthanized and uteri were isolated. Collected samples were either frozen in liquid nitrogen for biochemical analysis or fixed in 10% zinc formalin (Sigma-Aldrich, St. Louis, MO) or frozen in OCT compound (Tissue-Tek Sakura, Torrance, CA) for tissue analysis. Both paraffin-embedded and OCT-embedded tissues were sectioned at 7 μm.

### Western blot analysis

Tissue lysates were subjected to SDS-PAGE and Western blot analysis as described previously [41]. Primary antibodies used were GRP94 (1:5000, Enzo Life Sciences, Farmingdale, NY), PTEN (1:1000, Cell Signaling, Danver, MA) and GAPDH (1:5000, Santa Cruz Biotechnology, Dallas, TX).

### Tissue section staining

Immunostaining on paraffin-embedded or frozen tissue sections was performed as described previously [13, 42]. Tissue sections were incubated at 4°C overnight with primary antibodies against GRP94 (1:250, Enzo Life Sciences, Farmingdale, NY), cytokeratin 14 (1:400), p63 (1:200), Cyclin D1 (1:100), Ki67 (1:200) from Thermo Scientific (Fremont, CA), β-catenin (1:100, Santa Cruz Biotechnology, Dallas, TX), active β-catenin (1:50, Millipore, Billerica, MA), phospho-S6 (1:200), S6 (1:50), phospho-AKT (Ser 473, 1:50), AKT (1:200), phospho-Histone H3 (1:200) from Cell Signaling Technology (Danver, MA), cytokeratin 8 (1:100, Developmental Studies Hybridoma Bank, Iowa City, IA), α-Smooth Muscle Actin (1:2000, Sigma-Aldrich, St. Louis, MO), pan cytokeratin (1:50, Abcam, Cambridge, MA) and E-cadherin (1:50, BD Biosciences, San Jose, CA). Immunofluorescence was analyzed using a Zeiss LSM 510 confocal microscope with LSM 510 Version 4.2 SP1 acquisition software. Confocal images were acquired with 20X or 40X oil lens. Images were then processed with LSM Image Browser R4.2 and Adobe Photoshop CS5.

Staining for mucin by either mucicarmine or periodic acid-Schiff stain (PAS) followed by diastase digestion was performed by the clinical histology laboratory at University of Southern California using standard protocols.

### Cell lines and culture conditions

Human endometrial cancer cell lines AN3CA and ECC-1 have been described and authenticated by mitochondrial sequencing (7.1.11) [23]. Only short term cultures from the verified frozen stocks were used. The cells were maintained in Dulbecco's Modified Eagle media (Corning, Manassas, VA) supplemented with 10% fetal bovine serum (Omega Scientific, Tarzana, CA), 1% penicillin and streptomycin at 37°C in a humidified atmosphere of 5% CO_2_.

### WST-1 viability assay

Cell viability was assessed with the WST-1 reagent (Roche, Indianapolis, IN). Briefly, 10,000 cells per well were plated onto 96-well plates and the cell viability was measured by incubating each plate with 10 μl/well of WST-1 substrate for 2 hours and plates were red at a wavelength of 450 nm.

### RNA-seq dataset analyses

TCGA Level 3 RNA-seq data were downloaded from TCGA data access (https://tcga-data.nci.nih.gov/tcgafiles/ftp_auth/distro_ftpusers/anonymous/tumor/). The version number of RNA-seq dataset is IlluminaHiSeq_RNASeqV2_3.1.12. The data were all generated on Illumina HiSeq platform, mapped with the RSEM algorithm and normalized so that the third quartile for each sample equals 1000. Entrez gene IDs were used for mapping to genomic locations using GenomicRanges [43].

To compare the mRNA expression level of *Grp94* and *Pten* between normal and endometrial cancer samples, we log2 transformed the expression data [log2(RSEM+1)], and then performed a Student's *t*-test on gene expression between 24 normal and 177 tumor samples. In order to investigate the gene expression correlation between *Grp94* and *Pten*, Pearson correlation test was used on log2 transformed expression data.

### Statistical analysis

Statistical analysis was performed with the least significant difference (LSD) method, the 2-tailed Fisher's exact test or 2-tailed Student's *t*-test as indicated.

## SUPPLEMENTARY FIGURES


