# Supplementary material for: Glucose-regulated protein 94 deficiency induces squamous cell metaplasia and suppresses PTEN-null driven endometrial epithelial tumor development
Source: Oncotarget. 2016 Feb 17;7(12):14885–97. doi: 10.18632/oncotarget.7450 (PMC4924759; doi:10.18632/oncotarget.7450)
Supplement: Supplementary file 1 [file oncotarget-07-14885-s001.pdf]

SUPPLEMENTARY FIGURES

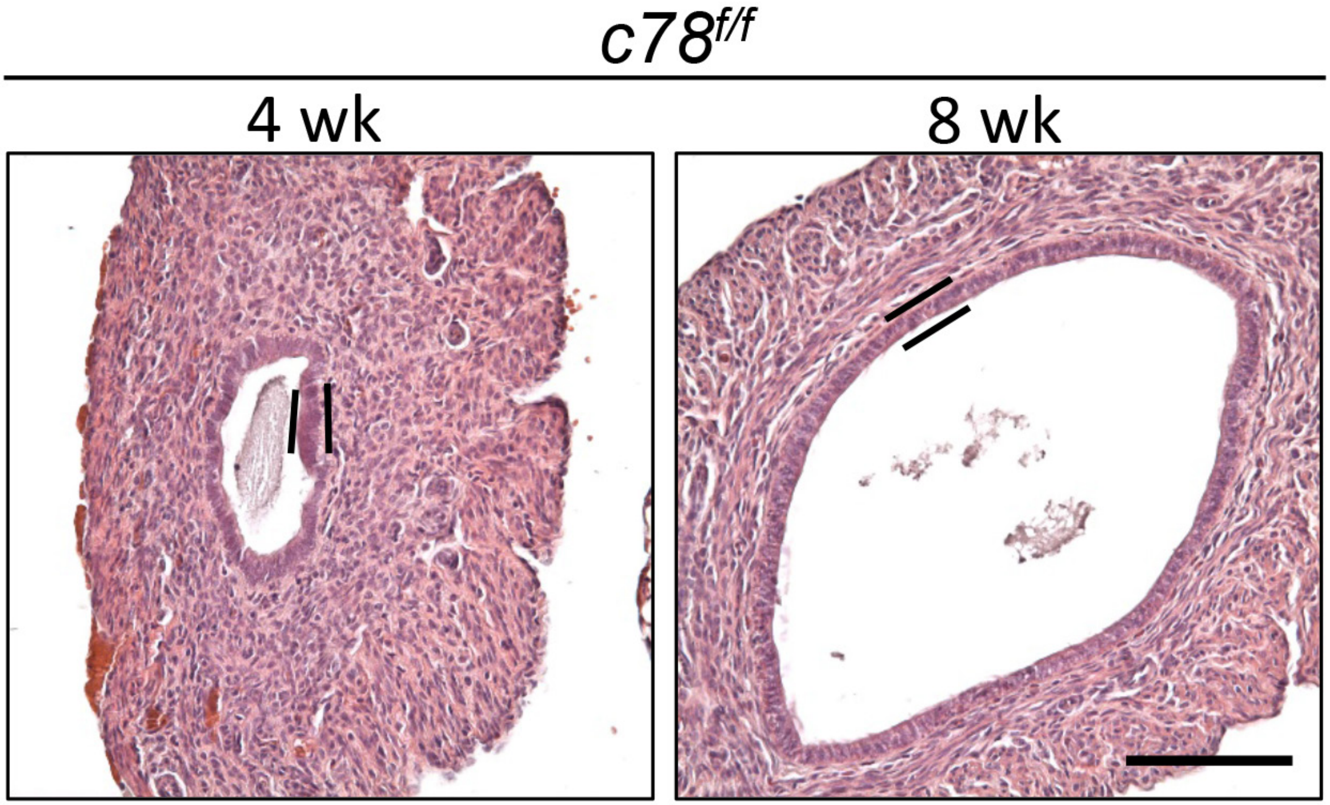

Supplementary Figure S1: GRP78 deficient uteri showed no SCM. H&E staining of *c78*$^{f/f}$ uteri at 4 and 8 weeks. Black bars denote a single layer of columnar epithelial cells. Scale bar, 100 µm.

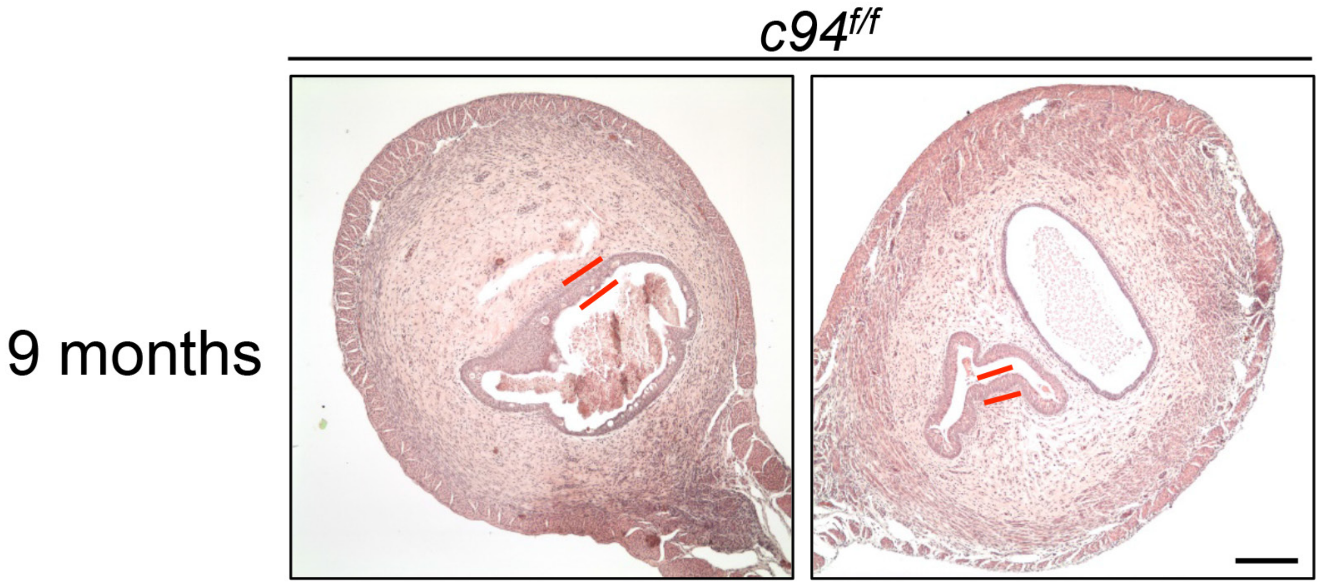

Supplementary Figure S2: *c94*$^{f/f}$ uteri showed SCM but no EAC at 9 months. H&E staining of *c94*$^{f/f}$ uteri at 9 months. Red bars denote SCM. Scale bar, 200 µm.

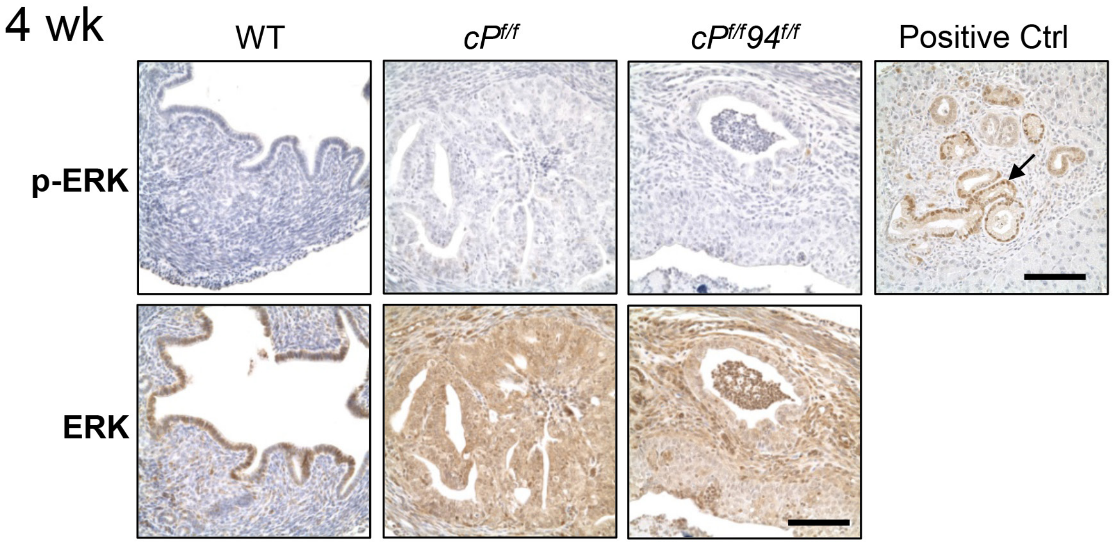

Supplementary Figure S3: *cP$^{f/f}$94$^{f/f}$* uteri showed no change on ERK activation. IHC of p-ERK and ERK in WT, *cP$^{f/f}$* and *cP$^{f/f}$94$^{f/f}$* mice uteri at 4 weeks with pancreas bearing Kras mutation as p-ERK positive control (arrow). The p-ERK (1:400) and ERK (1:100) antibodies were purchased from Cell Signaling Technology (Danver, MA). Scale bar, 100 µm.

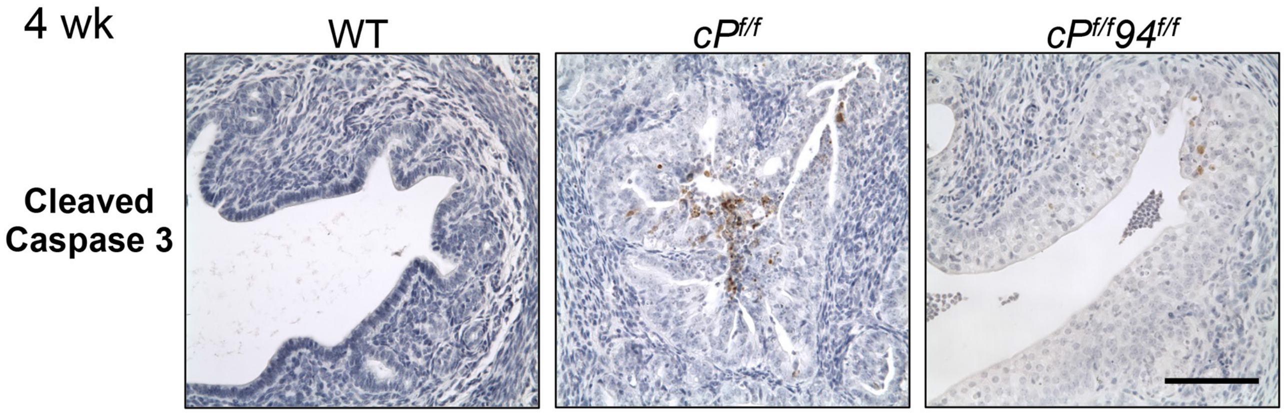

Supplementary Figure S4: *cP$^{f/f}$94$^{f/f}$* uteri show no increase in apoptosis. IHC of cleaved caspase-3 in WT, *cP$^{f/f}$* and *cP$^{f/f}$94$^{f/f}$* mice uteri at 4 weeks. The cleaved caspase-3 (1:250) antibody was purchased from Cell Signaling Technology (Danver, MA). Scale bar, 100 µm.

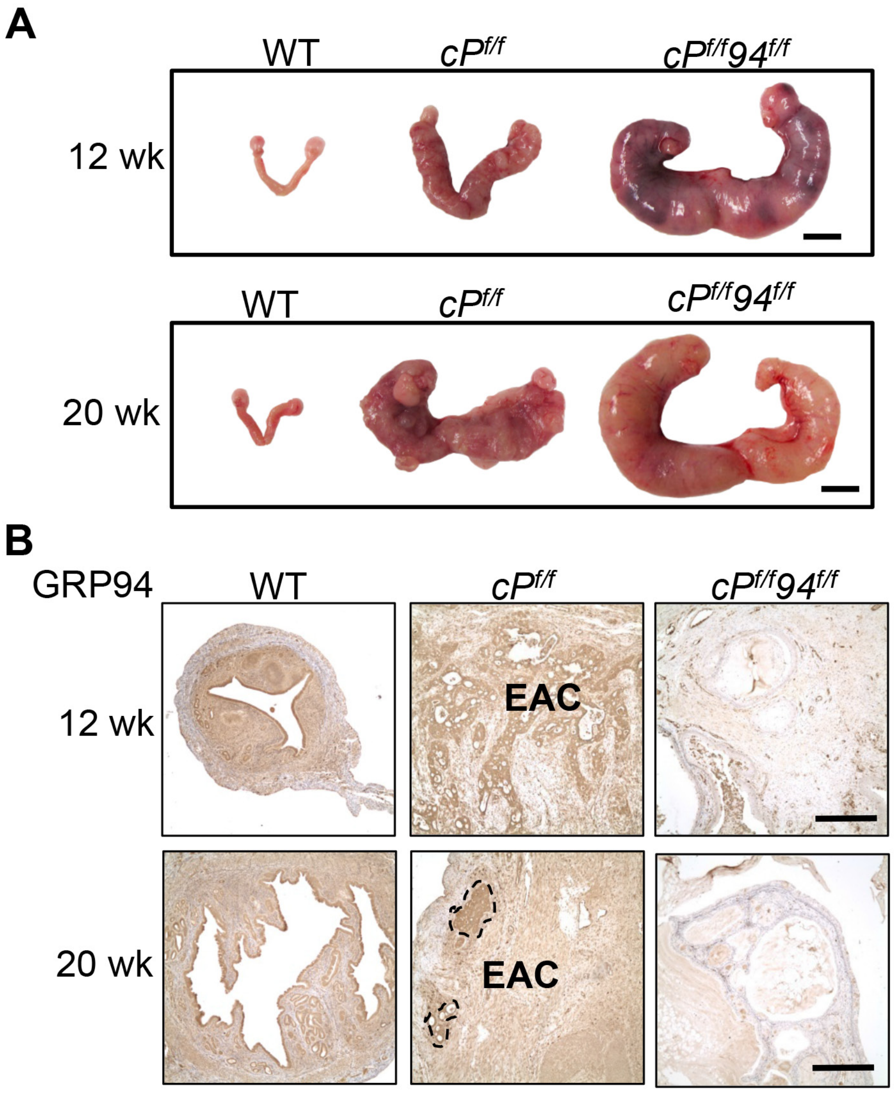

Supplementary Figure S5: Characterization of uteri at prolonged stage. A. Gross anatomy of WT, *cP*$^{f/f}$ and *cP*$^{f/f}$*94*$^{f/f}$ mice uteri at 12 weeks and 20 weeks. Scale bar, 0.5 cm. **B.** IHC of GRP94 in WT, *cP*$^{f/f}$ and *cP*$^{f/f}$*94*$^{f/f}$ uteri at 12 weeks (upper panel) and 20 weeks (lower panel). Scale bar: 400 μm.
